# Early Onset Colorectal Cancer: A Hypothesis

**DOI:** 10.1093/oncolo/oyad274

**Published:** 2023-09-27

**Authors:** Alfred I Neugut, Benjamin Lebwohl

**Affiliations:** Department of Medicine and Herbert Irving Comprehensive Cancer Center, Vagelos College of Physicians and Surgeons, New York, NY, USA; Department of Epidemiology, Mailman School of Public Health, Columbia University, New York, NY, USA; Department of Medicine and Herbert Irving Comprehensive Cancer Center, Vagelos College of Physicians and Surgeons, New York, NY, USA; Department of Epidemiology, Mailman School of Public Health, Columbia University, New York, NY, USA

## Abstract

The rising rates of early onset colorectal cancer are perplexing. This commentary discusses what may be causing the increase.

The public health community has been perplexed by rising rates of early onset colorectal cancer (CRC).^1^ Over the past 30-35 years, the incidence of CRC has been rising in populations below age 50 years, especially in groups aged 20-39 years. CRC incidence rates have doubled among those in the 20-39 age range and only slightly less among those in the 40-49 year age range as well.

Meanwhile, incidence rates of = in middle-aged and older populations have shown a decline, generally attributed to the increased utilization of colorectal screening, most prominently and effectively by colonoscopy and removal of precancerous polyps.^2^ Overall, CRC incidence rates have decreased, but due to the increasing incidence rate among the young and the decreasing incidence rate in those over age 50, younger patients now comprise almost 20% of all CRC patients in the United States.^3^

Policymakers at the National Cancer Institute and elsewhere have taken notice, and various investigators have devoted serious time and effort to seeking explanations, such as changes in lifestyle, genetic factors, or known high-risk medical conditions, such as inflammatory bowel disease. Investigators have also considered the possibility of new environmental agents; of course, to be responsible for the shift in incidence, any environmental factor would have to be widespread and strongly associated with CRC, but only in the young. Indeed, given that rising rates of CRC among the young have now been reported in most western countries,^4^ the environmental exposure would have to be ubiquitous among young people on a global scale.

What may be causing this increase in the incidence of CRC? The literature describes changes in several known risk factors for CRC, including increased obesity, reduced physical activity, reduced use of aspirin and other nonsteroidal inflammatory drugs, and dietary changes.^5^ These risk factors are present and operative across the age spectrum; they may, therefore, increase CRC incidence regardless of age. But perhaps, the increase in CRC incidence among older individuals is blunted and even negated by the widespread adoption of CRC screening and colonoscopic polypectomies in this population.

It is impossible to measure what the incidence of CRC in older individuals would have been had not screening been adopted. But there are settings outside of the United States and Europe, where screening would not interfere with the observation of incidence in the older age groups. These conditions are present in some countries of sub-Saharan Africa, where CRC incidence rates have been increasing along with affluence, obesity, and westernized diet, while physical activity has decreased. Population screening for CRC is not practiced in those countries.^6^

In sub-Saharan Africa, from 2010 to 2019, the age-standardized incidence rate of colorectal cancer has increased by 11% in the population as a whole.^6^ In Uganda, for example, from 1995 to 2015, the annual incidence rate of colorectal cancer in men younger than 50 years rose from 0.77 cases per 100 000 to 1.28 cases per 100 000, and in men 50-74 years from 15.48 to 23.66 cases per 100 000. A similar rise across age strata was present in women, and the same trend is observable in Kenya as well (Table 1). In contrast, in the United States, Germany, and other countries where colorectal cancer screening is routine and widely implemented, the rise is observable only in those age groups that are not yet eligible for screening (Table 1).^7^

**Table 1.** Changing colorectal cancer incidence (per 100 000 person-years) in the United States and Uganda from 1995-2015, by age group.

Age group, years	United States			Germany			Uganda			Kenya		
	1995	2015	Trend	1995	2015	Trend	1995	2015		1995	2015	Trend
Male												
15-49	10.27	12.57	↑	8.00	9.76	↑	1.40	2.39	↑	0.77	1.28	↑
50-74	204.04	154.79	↓	187.30	157.61	↓	31.96	43.36	↑	15.48	23.66	↑
Female												
15-49	8.75	10.66	↑	7.38	7.52	↑	0.97	1.55	↑	1.01	1.37	↑
50-74	139.72	103.56	↓	131.49	93.95	↓	17.24	28.13	↑	17.87	21.11	↑

Source^7^: https://vizhub.healthdata.org/gbd-results?params=gbd-api-2019-permalink/1167b532bc96643e8e2815ba027e46d2

There are myriad differences in environmental and genetic underpinnings in these countries. Still, the broad contrasts between the countries with screening programs and the countries without such programs support our hypothesis that the increase in CRC is not restricted to the young, but rather there is a slow-but-steady rise that encompasses all age groups, both those in the early-onset age groups and those in the middle and older age groups. This increase, which started in the late 1980s or 1990s, is on the order of 2% per year. When an increase of this magnitude is observed among those who are 20-29 years of age, whose baseline rate of CRC is about 0.5/100 000 per year, the observed incidence rate of CRC doubles in 10-15 years.^8^

If the same increase of 2% per year occurred in the 60-69 year age group, whose baseline incidence rate is approximately 100/100 000/year, the observed incidence rate would increase over 10 years by about 20%, to approximately 120/100 000, a much less dramatic change. The increase in screening, including the identification and removal of precancerous adenomas, over the same period might prevent many cancers that would otherwise increase the incidence rate, even driving it down below 100.^8^ As a result, not only would no increase be seen in those over age 50, but the incidence rate might actually decline, as it appears to have done in these older individuals.

What are the implications of this hypothesis? First, in our efforts to prevent early onset CRC, we must not neglect older-onset CRC. Although the rise in early onset colorectal cancer is a serious public health challenge, it should be seen as one aspect of the larger phenomenon of increased risk for CRC throughout the lifespan. Although the prevention of CRC in a person younger than 50 years yields a major gain in quality-adjusted life-years, a program for older age groups might prevent so many more cases of colorectal cancer that it might yield as many benefits, or even more.

Second, as we fund studies of environmental risk factors for CRC, these risk factors need not be exclusive to, or even predominantly affect, younger individuals. Obesity, reduced physical activity, declining aspirin use, and diet provide both an ample explanation and an opportunity for intervention for all age groups. The explanations are readily apparent, and not just present among young people or specific geographic regions.

Third, we can use this understanding to promote and implement more widespread screening in all eligible age groups. Guidelines have recently expanded to include individuals aged 45-49 years, but a push to expand our screening efforts in this younger population must not come at the cost of neglecting older individuals who are not yet screened.^9^ The fact that a potential rise in colorectal cancer incidence has been negated by screening older age groups is a triumph that should be built upon. In colorectal cancer epidemiology, the most important environmental exposure may be exposure to a colonoscope.
